# Perfusion Imaging with SPECT in the Era of Pathophysiology-Based Biomarkers for Alzheimer's Disease

**DOI:** 10.4061/2010/109618

**Published:** 2010-12-15

**Authors:** Markus Weih, Ümüt Degirmenci, Sebastian Kreil, Piotr Lewczuk, Daniela Schmidt, Johannes Kornhuber, Torsten Kuwert

**Affiliations:** ^1^Department for Psychiatry and Psychiatry, University of Erlangen-Nuermberg, Schwabachanlage 6, 91054 Erlangen, Germany; ^2^Clinic of Nuclear Medicine, University of Erlangen-Nuermberg, Schwabachanlage 6, 91054 Erlangen, Germany

## Abstract

SPECT allows registration of regional cerebral blood flow (rCBF) which is altered in a characteristic temporoparietal pattern in Alzheimer's Dementia. Numerous studies have shown the diagnostic value of reduced cerebral blood flow and metabolic changes using perfusion SPECT and FDG-PEPT in AD diagnosis as well as in differential diagnosis against frontotemporal dementia, dementia with Lewy bodies and vascular disease. Recently more pathophysiology-based biomarkers in CSF and Amyloid-PET tracers have been developed that probably have a higher diagnostic accuracy than the more indirect rCBF changes seen in perfusion SPECT. In the paper review, we describe recent advances in AD biomarkers as well as improvements in the SPECT technique.

## 1. Introduction

Memory problems in elderly patients are common and can be due to a wide range of conditions. Alzheimer's Disease (AD) accounts for a large amount of cases, especially when gradual onset and further cognitive problems like aphasia, apraxia, or reduced activities of daily life are present. AD originally has been a pathologic diagnosis. The “golden standard” of AD are comprehensive clinical criteria (like NINCDS-ADRDA, DSM-IV, or ICD-10). These criteria have been validated against pathology with a wide range of sensitivity (65%–100%) and specificity (65%–90%) [1]. Thus, during the last decades, imaging and cerebrospinal fluid (CSF) taps were then performed to rule out secondary memory disorders, for example, due to intracranial mass or infection (like syphilis). As a first approach to in vivo AD imaging, single proton emission computed tomography (SPECT) with perfusion tracers was developed and validated approximately 25 years ago. Perfusion SPECT shows a characteristic (nonvascular) pattern in parietal-temporal cortical areas that indirectly reflect the underlying spatial distribution of neurofibrillary and amyloid pathology. The coupling of fading neural activity and glucose metabolism to reduced regional cerebral blood flow (rCBF) allowed for the first time real albeit indirect “functional imaging” in neurodegenerative diseases using SPECT and later FDG-PET. More recently, advances in CSF protein assays and the development of specific amyloid-binding Positron Emission Tomography (PET) tracers allowed direct and pathophysiology-based tests and imaging. Here we would like to review the diagnostic value, accuracy, advantages, and disadvantages of perfusion SPECT in comparison to these more advanced CSF and PET biomarkers.

## 2. Diagnostic Value of Perfusion SPECT

Since 1991 more than 50 studies have been performed to compare the diagnostic value of SPECT (mostly ^99m^Tc-HMPAO) in AD versus normal volunteers, patients without neurodegenerative dementia (like depression) or other forms of dementia like frontotemporal dementia (FTD), dementia with Lewy bodies (DLB), or vascular dementia [2]. Taken together these studies clearly showed added value of SPECT for or against a clinical diagnosis of AD. A comprehensive review of Dougall et al. [3] identified 389 publications on this issue. After exclusion of nonrelevant studies using a methodology checklist the authors found 37 studies of clinical AD versus normal controls (comprising 1559 subjects) and 13 studies of AD versus nondemented control patients (1082 patients). The case-control studies with AD patients versus normal subjects were too heterogeneous to compare. The studies of clinical AD versus nondemented patients showed pooled sensitivities/specificities of 66% and 79%, respectively, yielding a diagnostic odds ratio of 8.2 [3]. In our own data base, we found sensitivities of 48%–60% [4] and a specificity of 90% [5]. Comparison of blinded SPECT versus a pathological confirmed diagnosis of dementia yielded SPECT sensitivity/specificity of 63%–86% and 73%–93% [6, 7]. Another important clinical question is the value of perfusion SPECT to predict progression from mild cognitive impairment (MCI) to AD. Devanand et al. [8] found a sensitivity of only 42% but a specificity of 82% for perfusion SPECT. Taken together these results suggest that SPECT has a sensitivity for AD that is most likely below the desired threshold of a valid biomarker, but a high specificity, making SPECT at least a useful diagnostic tool to rule out AD. In Table 1 the individual components of perfusion SPECT are displayed against other techniques.

## 3. Comparison of Perfusion SPECT and FDG-PET

The standard radionuclide in clinical PET centers is FDG (^18^F tagged to Deoxyglucose). FDG has long been the traditional method to measure secondary glucose metabolism changes downstream the neurodegenerative process in AD. Since cerebral metabolism and blood flow are coupled, there is a similar pattern of regional disturbed glucose metabolic rates (rCMRGlu) in the temporal-parietal cortex, less in the prefrontal area as mentioned before. The first description appeared in 1982 by Farkas et al. [9]. Since then, many other groups confirmed the diagnostic value of FGG-PET in AD. In a meta-analysis of 15 articles, Patwardhan et al. found a pooled sensitivity of 86% and a specificity of 86% [10]. FDG-PET and perfusion SPECT are the two most prominently available functional imaging methods available to clinicians worldwide. 

Direct comparison of both techniques in the same population has not been performed to our knowledge. When comparing the diagnostic accuracy based on meta-analytic data, there is some evidence for a higher sensitivity and specificity of FDG-PET when compared to perfusion SPECT.

## 4. Diagnostic Value of CSF-Based Biomarkers

Since the identification of Amyloid Precursor Protein (APP) in AD pathophysiology, there have been efforts to find appropriate APP-based CSF tests. However, initial assays measured all amyloid peptides and were not specific enough. After the pioneering work of Motter et al. in 1995 [11] who found a specific reduction of beta-amyloid peptide-42 in CSF, these results were then confirmed by many other groups. On average there is a reduction of beta-amyloid peptide-42 of approximately 40%–50%, and the tests have a sensitivity and specificity of approximately 85%. Other publications evaluated whether tau-pathology is also reflected in CSF changes. CSF levels of the microtubule-associated tau protein are typically increased (on average 2.5-fold) in AD and reflect neuronal damage [12]. Data from more than 35 single-center studies comprising more than 2500 AD patients and 1400 controls, meta-analyses, and consensus papers [13] are now available. When the specificity is set to 90% (which is a desirable value for biomarkers) total-tau has a sensitivity of app. 80% [14].

Hyperphosphorylation of tau (at position Threonine 181, 231 and Serine 199) occurs solely in AD and is therefore theoretically a more specific biomarker. However, studies showed large differences in sensitivity and specificity. A meta-analysis of 51 single-center studies by Mitchell [15] showed a pooled sensitivity of 78% and a specificity of 88% against patients without cognitive impairment. More recently, the different CSF markers have been combined [16] and the interpretation algorithms have been refined [17]. When evaluated in larger multicenter trials in patients with MCI, AD and nondemented patients or healthy subjects, the sensitivity of the combined CSF biomarkers (Amyloid peptide-42- and tau markers) to detect AD in MCI patients was then 85%–95% and the specificity 72%–83% [18, 19]. Other biomarkers like soluble APP are under investigation [20] and need to be validated. As a disadvantage it turned out in the CSF multicenter trials that there are larger than expected interlaboratory discrepancies [21].

## 5. Amyloid- and Tau-PET

The Pittsburgh Compound-B (^11^C-PiB) was the first of a new family of PET tracers that have shown to be of high diagnostic value in AD. Specific binding of PiB to brain Amyloid has been extensively studied in case-control studies and longitudinal observations since 2004 [22]. The area under the curve (AUC) in receiver operator characteristics (ROCs) for PiB is around 0.8-0.9 and can be improved to 0.94 when reserve variables like education, brain volume, gender, physical health, and medications are taken into account [23]. The main limitation for ^11^C-based PET tracers is their short half-life (appr. 20 minutes) which restricts their use to dedicated research sites with on-site cyclotrons and radiochemistry labs. ^18^F-based tracers have a longer half-life (appr. 110 minutes). The first ^18^F tracer for AD was FDDNP and developed to recognize both plaques and tangles. This could overcome the problem that some patients have extensive plaque formation but no dementia. The AUC for global rating of the FDDNP-PET could be up to 1.0 for AD versus control, 0.95 for MCI versus control and 0.98 for AD versus MCI. These promising results suggest that Amyloid-PETs or combined Amyloid-Tau-PETs may have a diagnostic value superior to other imaging tools like FDG-PET or volumetric MRI [24] in prevalent AD. Also, these tracers have been studies in MCI as a tool for early diagnosis but the results need to be validated in independent studies. Other studies with ^18^F-PET markers like Flutemetamol (PiB with ^18^F) are in phase II clinical studies [25]. Florbetapir (18F-AV-45) is the most widely studied ^18^F Amyloid imaging agent [26] and could receive FDA approval within next time. Other compounds like Florbetaben (ClinicalTrial NCT01020838) are in phase III clinical studies.

## 6. Differential Diagnosis: Vascular and Frontotemporal Dementia, Dementia with Lewy-Bodies

Although AD is the most prevalent dementia, several other neurodegenerative disorders have to be taken into account by the clinician. Vascular dementia is usually characterized by a past medical history with the presence of typical cardiovascular risk factors, stepwise deterioration, and vascular lesions on MRI or CT. Still, Perfusion SPECT has been used to discriminate vascular from primary neurodegenerative dementia. According to the meta-analysis of Dougall et al., the pooled weighted sensitivity and specificity against AD is 71%, respectively 76% [3].

Using clinical features alone, the differential diagnosis of frontotemporal dementia (FTD) and Dementia with Lewy-Bodies (DLB) is sometimes difficult. FTD is a heterogeneous disease and in contrast to AD no established specific and validated biomarkers are available. In the above-mentioned review of Dougall et al. 2004 [3], the authors found a pooled sensitivity and specificity of SPECT for discriminating AD from FTD of 72% and 78%, respectively The diagnostic odds ratio was 8.4 and is in the same range as that for AD versus control. In pathologically confirmed FTD cases, McNeill et al. found a sensitivity of bilateral frontal lobe CBF reduction of 80% and a specificity of 81% versus AD [27]. In a larger sample of FTD patients, Mendez et al. found a sensitivity/specificity of 91% and 75% and a negative predictive value of 90% [28]. These results suggest that SPECT provides useful additional information in discriminating AD from FTD. Some patients with Dementia with Lewy bodies (DLB) have prominent cognitive deficits but MRI usually does not show the characteristic global atrophy as seen in AD. Here several nuclear medicine techniques may be of advantage like assessment of occipital hypoperfusion alone [29] or in combination with MRI [30]. If symptoms of Parkinson's disease are predominant in DLB, Iodine-123 FP-CIT SPECT is of diagnostic value [31]. The characteristic sympathetic denervation in DLB can be assessed using Iodine-123-MIBG in a very specific manner [29]. Given the fact that SPECT scanners are available in many larger hospitals, the differential diagnosis of FTD and DLB against AD with SPECT is feasible and clinicaly relevant, since antidementive drugs like cholinesterase-inhibitors are unlikely to be of therapeutic benefit in FTD, but are approved for AD, Parkinson's disease dementia (PDD) and are beneficial in DLB (positive therapeutic studies, but off-label use).

## 7. Technical Improvements

The image acquisition technique in SPECT is prone to signal degradation due to different physical phenomena like depth-dependent blurring, photon attenuation and scattering.

SPECT images are evaluated similar to other imaging techniques like MRI or CT, inferring bias due to subjective image interpretation. Technical limitations in the past made iterative image reconstruction necessary [33]. In recent years, considerable advances have been made in this area like the introduction of hybrid SPECT/CT devices (for simultaneous registration of anatomy and function) and optimized, fast imaging reconstruction hard- and software. These advances now allow improved calibration and quantification of SPECT images. The coregistration of SPECT images with structural imaging and advances in automated anatomical labeling (AAL) opened the possibility to identify voxels of interest (VOI) which increases the originally limited spatial resolution of SPECT ultimately leading to better discrimination of AD patients. In addition, Pagani et al. [34] as well as Merhof et al. [35] has recently shown that rCBF patterns together with anatomical information can be subjected to principal component analysis (PCA) resp. multivariate analysis, which allows to draw further conclusion about disturbed functional connectivity between brain regions in dementia. Perfusion imaging using arterial spin labeling MRI is a standard technique in cerebrovascular disease and has been applied to AD diagnosis and differential diagnosis to FTD with promising results [36, 37] but the exact diagnostic value parameters like sensitivity, specificity have not yet been delineated in detail.

## 8. Areas of Uncertainty

It is still unclear whether the “AD pattern” in perfusion SPECT merely reflects cortical neurodegeneration or the secondary cholinergic deficit following degeneration of the basal nucleus of Meynert. Studies that both investigated CSF biomarkers and SPECT showed no correlation [5, 38]. Simultaneous registration of the cholinergic deficit (e.g., using Nicotinic 123I-5IA-85380 SPECT [39]) and perfusion SPECT or Amyloid-PET with perfusion SPECT could help to resolve this issue.

In perfusion SPECT usually lobar hypoperfusion is registrated. More recently, MRI techniques have shown that there is also compensatory hyperperfusion in AD and FTD [36]. Possible underlying mechanisms are partial deafferentation or a cognitive reserve mechanism. It would be interesting to confirm these results in perfusion SPECT, resp. to explore the diagnostic value of hyperperfusion in correlation to reserve variables.

## 9. Conclusions

Perfusion SPECT and FDG-PET are metabolic based and were the first methods of “functional” brain imaging in Alzheimer's disease showing hypoperfusion in the temporo-parietal regions with the highest load with plaques and tangles in postmortem brains with AD.

This has lead to a widespread use of perfusion SPECT and FDG-PET as a diagnostic tool in AD. Since specificity exceeds sensitivity the diagnostic value of perfusion SPECT to rule out AD usually is higher than to confirm it. However, accuracy of any diagnostic tool is critically dependent on disease severity and the population under investigation. In addition, perfusion SPECT is useful in discriminating vascular dementia, FTD, and DLB in the absence of validated specific biomarkers for these conditions. More recently pathophysiology-based CSF-biomarkers, especially beta-amyloid peptide-42 and tau protein, have been investigated with a diagnostic accuracy that seems to be superior to perfusion SPECT and FDG-PET. In addition, due to their direct reflection of the underlying process, CSF biomarkers (alone or in combination) are also suited for early diagnosis, for example, in patients with MCI, where the diagnostic value for SPECT is even lower than in prevalent AD. In contrast to CSF assays, PET biomarkers have the advantage of showing the anatomic distribution of pathology and were first available for amyloid plaques (^11^C PiB). Now, there is development of longer-lasting ^18^F PET tracers as well first evidence that plaques and tangles can be imaged with a single PET compound, but the results have to be confirmed in independent studies.

## Figures and Tables

**Table 1:** Perfusion SPECT versus biomarkers in AD.

Method	FDG-PET	Perfusion-SPECT	CSF	Amyloid-PET
First description	Farkas et al., 1982 [9]	Launes et al., 1991 [2]	Motter et al., 1995 [32]	Klunk et al., 2004 [22]
Diagnostic accuracy Sensitivity Specificity Meta-analysis	[10] 86% (CI: 76%–93%) 86% (CI: 72%–93%) Yes	[3] 66% (CI: 62%–69%) 79% (CI: 75%–83%) Yes	[13, 15] 80%–85% 85%–90% Yes	[23] appr. 85%–100%% appr. 85%–100% No
Clinical availability	Academic centers	Greater hospitals	Theoretically everywhere	Academic centers
Equipment	Cycloton	Gamma-camera	ELISA reader	Cycloton
Cost Estimation	>1000 EUR	<300 EUR	<200 EUR	>2000 EUR
Expertise for result interpretation	High	High	High	High
Treatment options if test positive	MCI: negative	MCI: negative	MCI: negative	MCI: negative
	AD: positive	AD: positive	AD: positive	AD: positive
		DLB: off-label FTD: negative		

CI: 95% confidence interval. MCI: mild cognitive impairment.
